# Schwartz Centre Rounds: a new initiative in the undergraduate curriculum—what do medical students think?

**DOI:** 10.1186/s12909-016-0762-6

**Published:** 2016-09-22

**Authors:** Faye Gishen, Sophia Whitman, Deborah Gill, Rhiannon Barker, Steven Walker

**Affiliations:** 1 Faculty of Medical Sciences, University College London Medical School, 74 Huntley Street Building, London, WC1E 6AE UK; 2 St Gilesmedical, The Vestry House, 60 St Giles High Street, London, WC2H 8LG UK; 3 Point of Care Foundation, 11-13 Cavendish Square, London, W1G 0AN UK

**Keywords:** Education, Medical student, Teaching, Undergraduate, Curriculum, Schwartz centre rounds, Resilience, Compassion, Burnout, Stress

## Abstract

**Background:**

Training to be a doctor and caring for patients are recognized as being stressful and demanding. The wellbeing of healthcare professionals impacts upon the wellbeing and care of patients. Schwartz Centre Rounds (SCRs), multidisciplinary meetings led by a trained facilitator and designed for hospital staff, were introduced to enhance communication and compassion, and have since been widely adopted as a way of fostering compassion. The continuum of education suggests that medical students need to develop these attributes in conjunction with resilience and maintaining empathy. The benefits of SCRs in fostering this development in medical students is unexplored.

The objective of this study was to examine the potential of SCRs within the undergraduate curriculum.

**Methods:**

Two student–focused SCRs were piloted at a major medical school. The sessions were based on the current format implemented across the US and UK: a presentation of cases by a multidisciplinary panel followed by an open discussion with the audience. Participants were asked to complete an evaluative questionnaire immediately following the sessions. Seven students took part in a focus group to explore their views on the SCR. Data sets were examined using descriptive statistics and thematic analysis.

**Results:**

Feedback was obtained from 77 % (258/334) Year 5 and 37 % (126/343) Year 6 students. Mean student ratings of the session on a five-point scale, where 1 = poor and 5 = exceptional, were 3.5 (Year 5) and 3.3 (Year 6). Over 80 % of respondents either agreed or strongly agreed that the presentation of cases was helpful and gave them insight into how others feel/think about caring for patients. Eighty percent said they would attend a future SCR and 64 % believed SCRs should be integrated into the curriculum. Focus group participants felt SCRs promoted reflection and processing of emotion. Students identified smaller group sizes and timing in the curriculum as ways of improving SCRs.

**Conclusion:**

Students were positive about SCRs, preferring them to their current reflective practice assignments. Whether this results in sustained benefits to trainee doctors is yet to be explored. Consideration is given to overcoming the challenges that were encountered, such as optimal timing and participation. Staff training and costs are potential obstacles to adoption.

**Electronic supplementary material:**

The online version of this article (doi:10.1186/s12909-016-0762-6) contains supplementary material, which is available to authorized users.

## Background

A substantial proportion of newly-qualified doctors consider themselves under-prepared for the role; in particular being able to cope with the physical and emotional demands of medical practice [1]. Systematic reviews have reported a significant decline in empathy as students progress through medical school, and a high prevalence of burnout among medical undergraduates [2, 3]. More needs to be done to instill students with appropriate values and habits to sustain a long and demanding career in medicine. The psychological wellbeing of medical students impacts not only themselves but also has important implications for their future patients [4]. Guidelines from the General Medical Council (GMC) recommend introducing measures during training to enhance ’resilience, professionalism and employability’ [5].

Key questions remain as to how medical schools can foster resilience and maintain compassion during medical training with an ever-increasing focus on ‘cure’ and use of technology. In postgraduate medicine in the UK, a number of initiatives have been introduced which aim to promote compassion and empathy in the individual and their institution [6]. Balint groups—facilitated small groups of HCPs discussing emotionally challenging cases—now widely used in primary care, are claimed to increase job satisfaction and prevent burnout [7]. By comparison, ‘Compassionate Conversations’ is a small initiative aimed at hospital staff as diverse as porters and chief executives to promote staff engagement and reflection [8].

Models of self-reflection share the premise that examining past experiences and assimilating what is learnt from this will act as a guide in future situations. Evidence on the efficacy of reflective practice is sparse, with many studies being observational and rarely including a comparison group. A literature review identifying 29 articles concerning reflective practice in HCPs found that when reflection was shared with colleagues it was more effective [9]. The authors posit that this is due to the multiple perspectives and insight a group provides. Although the authors found no evidence regarding reflection improving patient care, they conclude that “awareness of uncertainty and validation of assumptions are part of reflective practice and might theoretically have the potential to improve patient care”.

Schwartz Centre Rounds (SCR) are gaining in popularity and may be more universally applicable than other models of reflective practice [10]. Originating in the United States, the program was first developed by the Schwartz Center for Compassionate Healthcare, to help to bring compassion to patient–caregiver interactions. Initially the focus was on staff providing acute care, but since their origins in 1995 they have spread both geographically and into a range of clinical settings. SCRs were introduced into the UK in 2009 and now operate across 125 different organisations in a range of diverse settings, including mental health and community trusts [11].

Current SCRs consist of a forum for clinical and non-clinical staff from all backgrounds and levels within a healthcare organisation to come together and explore the impact that their job has on them. A team who has cared for a patient tell their stories and this is followed by an open, confidential audience discussion, exploring issues that have arisen. The focus is not problem solving— rather, it is dedicated time for reflection and a safe place to voice feelings not often shared. Participants have reported increased insights into social and emotional aspects of patient care and increased feelings of compassion towards patients and colleagues [10, 12].

It is postulated that fostering self-care and reflection promotes better patient-centred care as a consequence. SCRs were mentioned in the *Francis Report* as valuable means of developing a compassionate culture in healthcare [13].

This study examines the applicability of SCRs to medical schools, identifying whether it is possible to extrapolate the reflective, supportive culture that they foster to the undergraduate setting. Reflective groups based on the SCRs model have been held at a small number of American Medical and Health Allied Science Professional schools. Early results are encouraging [14] but it is unknown whether these models could translate into a UK education culture. Moreover, there is no standard model for the ‘what’ ‘when’ or ‘how’ SCRs might be conducted within a training environment, nor any indication of how sustainable such initiatives would be.

## Methods

### Participants and setting

Two 60-minute pilot SCRs were undertaken at a UK medical school. The first SCR involved Year 5 medical students and was integrated into an introductory week during the start of their academic year. A multidisciplinary panel of doctors and nurses presented a series of stories with the theme *“A Patient I Will Never Forget”*. Subsequently, two facilitators who had attended training on leading SCRs led a confidential whole group discussion lasting 35 minutes.

The second pilot SCR took place with Year 6 students and followed the same format as the first. The session formed part of a teaching day just prior to their final examinations. The topic was *“In at the Deep End”*. This focused on the transitioning from being a medical student to a qualified doctor and included a medical student member on the multidisciplinary panel.

In line with the existing SCR model, students’ attendance at the pilot SCRs was encouraged but not mandated.

### Data collection and measures

A simple, evaluative questionnaire based on the feedback form employed by the Point of Care Foundation was administered immediately after each student SCR (Additional file 1). Additional free-text comments were also collected. The anonymised questionnaire was kept brief to encourage completion and comments. Attendance at the session and completion of the anonymous questionnaire was indicative of consent to participate. Responses involved a combination of “Yes” or “No” answers, a five-point Likert Scale or a score out of 5 where the highest score was “exceptional” and the lowest “poor”.

Following the first SCR, seven students from Year 5 volunteered to participate in a focus group. This took place 10 days after the SCR. Two researchers posed open questions using a semi-structured approach to explore students’ views about the session and their experiences of compassion fatigue and burnout. Students who volunteered for the focus group gave their verbal consent to participate and for the session to be recorded. Students’ input remained anonymous.

### Analysis

Quantitative data was imported into Microsoft Excel and Stata 13. Percentages and mean scores were calculated for questions employing a Likert scale or score, and percentages for dichotomous responses. To compare views between years, the responses were divided into nominal categories and analysed using Chi-squared and Fisher’s exact tests when the sample sizes were small.

The free text responses provided by both groups were entered into Excel and were coded and then reviewed for emerging themes. A third researcher who was experienced in qualitative analysis methodology read the responses, ratified the coding process and reviewed the development of themes.

The transcribed contents of the focus group meeting was also explored using a thematic analysis approach [15]. The data was independently reviewed by two members of the research team. From this exercise a number of codes were generated from the transcript. Following a second review of the transcript, a matrix was constructed that allowed the initial codes to be collated into themes. The themes were then reviewed and refined by a third researcher as described above. A member of the focus group was asked to assess if the themes were a true reflection of the discussions that took place.

## Results

### Uptake

Of the 334 Year 5 students invited to the first SCR, 258 students attended (77 %) and 247 of these participants (96 %) answered the questionnaire. Of the 343 Year 6 students invited to the second pilot, 180 attended (52 %) and 126 of these participants (70 %) answered the questionnaire. Available case analysis was used to analyse the data, as some students omitted answers to a small proportion of the questions.

### Quantitative assessment

**Fig. 1:**
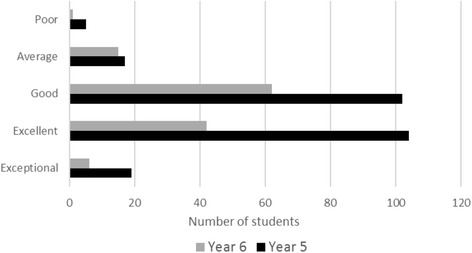
Year 5 and Year 6 responses to rating of SCR

**Fig. 2:**
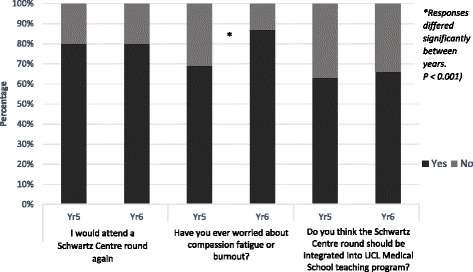
Year 5 and Year 6 responses to yes/no questions

**Fig. 3:**
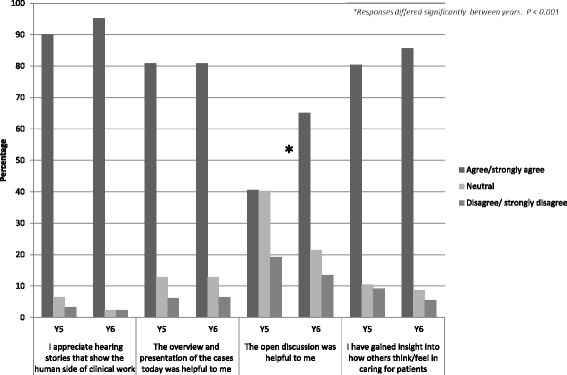
Year 5 and Year 6 responses to Likert scale questions

When asked to rate the SCR (five-point scale where 1 = poor and 5 = exceptional), both SCRs received a similar overall mean rating score: Year 5’s score was 3.5 (*n* = 247) and Year 6’s score was 3.3 (*n* = 126) (Fig. 1). The difference in rating between years was non-significant (NS). Combining students from both years who responded to the questionnaire, 80 % (292/365) said they would attend a future SCR and 64 % (235/366) thought that SCRs should be integrated into the medical school curriculum (Year 5: Year 6 *p* = NS) (Fig. 2). With regard to storytelling as an educational approach, 92 % (340/370) either agreed or strongly agreed that they appreciated hearing stories demonstrating the human side of medicine (Year 5: Year 6 *p* = NS) (Fig. 3). Similarly, 82 % (301/366) of students either agreed or strongly agreed that attending the SCR (296/366) gave them insight into how others feel/think about caring for patients (Year 5: Year 6 *p* = NS).

Eighty one percent (296/366) of students either agreed or strongly agreed that the presentation of cases was helpful (Year 5: Year 6 *p* = NS). By comparison, more students in the final year either agreed or strongly agreed that the open discussion at the end of the SCR was helpful (Year 5: 41 % [97/239]; Year 6: 65 % [82/126] *p* < 0.001). With regard to compassion fatigue, 69 % (165/240) of Year 5 students reported being worried about compassion fatigue or burnout, compared with 87 % (108/124) of Year 6 students (*p* < 0.001) (Fig. 2).

### Qualitative analysis

The commonest themes to emerge from an analysis of the free responses are shown in Table 1. There was overlap between these and the themes to emerge from the focus group.

**Table 1:** The four commonest themes to emerge from an analysis of the free responses provided by Year 5 and Year 6 students

Commonest themes	Year 5	Year 6
1	Students felt inhibited participating in front of a large group of their peers. There was a preference for holding SCR in smaller groups	Concerns about starting Foundation Training raised in the presentation
2	Value of raising emotive issues i.e. empathy, reassurance, vulnerability, importance of team building	Holding the SCR at a more suitable point within the curriculum i.e. not just before ‘finals’
3	Benefits of engendering personal reflection	Benefits of reflection
4	Participants found attending a SCR to be an emotional experience	Preference for holding SCR in smaller groups

Analysis of the focus group transcript revealed seven, related themes. These fell under two broad headings. The first was “Feelings towards the SCR”, comprising: 1, Responses to the session; 2, Size of the audience; 3, Comparison with current reflective practice; 4, Post-event peer discussions. The second category was “Psychological aspects of SCR”. Here three themes emerged: 1, The Psychological pressures of medicine; 2, How the session encouraged a positive processing of emotion; 3, Sharing of personal stories between health care professionals.

### Feelings towards SCR

All members of the focus group expressed a positive attitude towards the session they attended. They tended to favour the format over the current reflective practice in the curriculum, believing that it stimulated more self-reflection than having to submit an essay:

*Schwartz Rounds are a better way to get people to reflect than the writing tasks that we are required to do throughout the year—these tasks are just an obligation.*

Concerns were voiced regarding the size of audience; with several students reporting that they did not feel confident sharing their stories in front of such a large group:

*It is very hard to contribute and put your hand up in front of 300 people. I could have contributed quite a few things but I wasn’t going to say anything in front of 300 people.*

Although students may not have shared their stories in front of the audience, they found that it engendered beneficial discussions with their peers afterward the event:

*One of the most useful things for me was going home and talking about it.*

### Psychological aspects of the SCR

Several of the students spoke about the psychological pressures of working in medicine. Much of this was focused around how the expressing of emotion is often suppressed among medics:

*I think how you deal with your personal situation is something that’s really swept under the carpet in the medical profession.*

Students felt that the SCR allowed these emotions to be voiced and appreciated hearing healthcare professionals sharing their personal stories. Several students commented that listening to a transplant surgeon speak about harvesting an organ from a child highlighted a “more human” side to senior staff that might not have otherwise been exposed:

*It was nice to see that someone so far into his career still has those heart-wrenching moments.*

Further, hearing HCPs discussing how different cases had affected them emotionally demonstrated to the students that it was normal and acceptable to have these feelings. Students reported that the SCR enabled them to acknowledge and process the emotions they experienced when faced with some of the difficult issues encountered while on placements:

*It helps knowing that whilst being professional you can still have feelings and get upset about things.*

## Discussion

### Interpretation of the study results

This study reports the findings of an exploration of the potential of SCRs from the student participants’ viewpoint. As far as the authors are aware, this is the first time that the application of dedicated SCRs for medical students outside of the United States has been reported. Medical students are often invited to hospital rounds, but this particular session is a new innovation in the UK medical setting. The sessions were predominantly well received by the students, and important insights and understandings seem to have been fostered.

The attendance for the Year 5 SCR was more than twice that of students in thein the final year. The first session was held at the beginning of Year 5 during an introductory course when students are likely to have been more receptive. By comparison, the timing of the second pilot shortly before the Year 6 final examinations was not ideal, though the topic discussed was highly relevant to the training stage of cohort.

It is disappointing that both pilots did not receive an overall rating higher than 3.5 and only 64 % agreed that SCRs should be incorporated into the curriculum. This needs to be balanced with the finding that that 80 % of students said that they would attend a future SCR and that this sort of intervention is relatively intangible and aimed at attitudinal and personal outcomes rather than measurable gains in knowledge or skills.

Two differences were found between the responses of Year 5 and the Year 6 participants. More students in the final year valued the open discussion at the end of their session. This may be related to the second difference, namely greater concern about compassion fatigue and burnout than their junior colleagues with relatively less clinical exposure. Research suggests that 80 % of medical students experience at least one episode of distress during training, including burnout, low quality of life, depression, sleepiness and stress [16]. Increased levels of stress among medical students is associated with a reduction in empathy, a finding reported to be ameliorated by higher levels of social support. [17] Students who engaged with their peers and sought their support were found to be more resilient and more satisfied with their life [18]. SCRs may encourage peer support, as demonstrated by the emergent theme from the focus group analysis that the SCR led to peer-peer discussions after the session.

Other contributory factors for fewer Year 5 students finding the open discussion helpful might have been the choice of topics i.e. too ‘soft’ when their focus is on facts and the absence of someone on the panel they could identify with i.e. a fellow medical student.

The size of the audience was an issue raised after both SCRs and during the focus group. Students commented that the large audience inhibited them from sharing their own stories and contributing to the discussion. This issue has not been raised in studies of SCRs for hospital staff [10, 12] but is perhaps more of a difficulty for students for whom the audience consists of contemporaries. A possible solution may be to ask students to first share their stories with those around them. An attenuated version of SCRs called “Compassionate Conversations” that are held in less formal settings and where staff were asked to first share their experiences with a neighbour, before joining an open discussion has been trialed in a UK hospital and received positive feedback, with 64 % of participants rating the session nine or 10 out of 10 [8].

Alpert Medical School has adapted SCRs for medical students by creating smaller discussion groups [14], these have proven popular with students. Future UK SCRs for students may benefit from smaller group sizes, less formal settings and trialing sharing contributions with neighbours prior to sharing with a larger group.

To date, no guidance exists on the optimal number of rounds for achieving maximum benefit to staff and patients. However, Lown and Manning observed that the more SCRs staff attended, the greater the perceived impact they had upon feelings of being supported, less stressed, and less isolated [10]. Considering that a theme among the Year 6 free responses was worry about embarking on Foundation Training, students may benefit from more regular sessions.

### Limitations, cost and future research

This study does not set out to ‘prove’ that SCRs should be incorporated into all undergraduate curricula. It may be that had there been universal attendance or involvement of Years 1–4 students the result would have been different. Similarly, data from two pilot SCRs gathered immediately after delivery are insufficient to be generalizable. However, the number of responses received and concurrence of subjects raised by students in their free text contributions and during the focus group do provide evidence as to the potential benefits of SCRs and encouragement for their further development within the curriculum.

Conducting SCRs has a cost and time implication. In the UK, SCRs are licensed through the Point of Care Foundation (POCF). An initial two-year contract which includes training of a Facilitator and Clinical lead and support through a buddying arrangement with a neighbouring site is likely to cost around £5,000 and £1000 for membership thereafter. The course leads need time to prepare for the session. All of these considerations may impact on a school’s ability to incorporate formal SCRs in their curriculum.

Further research into the merit of SCRs for medical students is warranted. This could include a more in-depth analysis of the psychological demands placed upon medical students and the impact of SCRs on their subsequent interactions with patients.

## Conclusions

SCRs have been taking place in hospitals in the US and UK for several years and are credited with creating a culture than encourages interdisciplinary communication and fosters resilience among HCPs. The first UK pilots of SCRs for medical students received positive feedback and the majority would welcome their integration into the curriculum. Medical students are frequently ‘examination-centric’ in their approach to undergraduate training, but should be encouraged to reflect on their practice, something they will be required to do throughout their future medical career. Further consideration is required around timing and format to optimise participation and enhance benefits to students. Training of staff and costs present practical issues regarding embedding and sustaining SCRs into the medical school culture.

## Additional file

Additional file 1: Student Questionnaire. (DOCX 13 kb)

## Abbreviations

GMC: General medical council
HCP: Health care professional
POCF: Point of Care Foundation
SCR: Schwartz Centre Round
UK: United Kingdom
