# Supplementary material for: Schwartz Centre Rounds: a new initiative in the undergraduate curriculum—what do medical students think?
Source: BMC Med Educ. 2016 Sep 22;16:246. doi: 10.1186/s12909-016-0762-6 (PMC5034622; doi:10.1186/s12909-016-0762-6)
Supplement: Additional file 1: — Student Questionnaire. (DOCX 13 kb) [file 12909_2016_762_MOESM1_ESM.docx]

Schwartz Centre Rounds

Thank you for attending the Schwartz Centre Rounds today. The goal of the Schwartz Centre Rounds is to provide a multidisciplinary forum where staff discuss issues they face in providing compassionate care to patients. Please take a minute to answer these questions. UCL Medical School and Point of Care Foundation will use your response and comments to develop future Schwartz Centre Rounds.

Please respond to the following statements by checking the box that most reflects your opinion of today’s Schwartz Centre Round.

|  |  | **Disagree Completely** | **Disagree Somewhat** | **Neither Agree nor Disagree** | **Agree Somewhat** | **Agree Completely** |
| --- | --- | --- | --- | --- | --- | --- |
| 1, I appreciate hearing stories that show the human side of clinical work |  | □ | □ | □ | □ | □ |
| 2, The overview and presentation of the cases today was helpful to me |  | □ | □ | □ | □ | □ |
| 3, The open discussion was helpful to me |  | □ | □ | □ | □ | □ |
| 4, I have gained insight into how others think/feel in caring for patients |  | □ | □ | □ | □ | □ |
|  |  |  |  |  |  |  |
|  |  | **Yes** |  | **No** |  |  |
| 5, I would attend a Schwartz Centre round again |  | □ |  | □ |  |  |
| 6, Have you ever worried about compassion fatigue or burnout? |  | □ |  | □ |  |  |
| 7, Do you think the Schwartz Centre round should be integrated into UCL Medical School teaching program? |  | □ |  | □ |  |  |
|  |  |  |  |  |  |  |
|  |  | Poor | **Average** | **Good** | **Excellent** | **Exceptional** |
| 8, Please rate today's Schwartz Centre Round |  | □ | □ | □ | □ | □ |
|  |  |  |  |  |  |  |

Please feel free to make any further comments below including how the Schwartz Centre Round made you feel:
